# The estrogen–brain interface in neuroinflammation: a multidimensional mechanistic insight

**DOI:** 10.3389/fnagi.2025.1671552

**Published:** 2025-09-01

**Authors:** Jie Lu, Tie-Jun Xian, Cheng-Jun Li, Yang Wang

**Affiliations:** ^1^Department of Respiratory and Critical Care Medicine, The First People’s Hospital of Shenyang, Shenyang Brain Hospital, Shenyang, China; ^2^Department of Pleurisy, The Tenth People’s Hospital of Shenyang, Shenyang Chest Hospital, Shenyang, China

**Keywords:** estrogen, neuroinflammation, estrogen receptors, mitochondrial function, DNA repair, gut–brain axis

## Abstract

Neuroinflammation plays a dual role in the central nervous system, offering protection in acute phases but contributing to chronic damage in neurodegenerative diseases. Estrogen, traditionally recognized for its reproductive functions, exerts extensive neuroprotective effects by modulating neuroinflammatory processes across multiple levels. This review explores the actions of estrogen through its receptors in astrocytes, microglia, and neurons, emphasizing its regulation of signaling pathways such as PI3K/Akt, NF-κB, and WNT/*β*-catenin. Estrogen also enhances mitochondrial function, promotes DNA repair, and interacts with the gut microbiota to influence systemic inflammation. Furthermore, sex-specific responses to 17*α*-estradiol highlight the importance of hormonal context. Together, these findings underscore estrogen’s potential as a multifaceted modulator of neuroinflammation and provide insight for precision therapeutic strategies.

## 1 Introduction

Neuroinflammation is a response initiated by specialized cells following brain injury, aiming to restore tissue homeostasis (Shi and Yong, 2025). It involves multiple cell types, including neurons, microglia, astrocytes, and endothelial cells. During the inflammatory response, disruption of the blood–brain barrier (BBB) often facilitates the infiltration of peripheral immune cells, such as monocytes/macrophages and lymphocytes, into the central nervous system (CNS) (Candelario-Jalil et al., 2022). In the acute phase, neuroinflammation is generally beneficial, contributing to the resolution of injury and enhancing the brain’s defense against pathogens and other insults. However, in many neurological disorders—including Alzheimer’s disease, Parkinson’s disease, and multiple sclerosis—neuroinflammation becomes exaggerated and chronic. Prolonged or excessive activation of microglia and astrocytes leads to overproduction of pro-inflammatory cytokines and chemokines, resulting in synaptic dysfunction, neuronal damage, and ultimately irreversible cognitive and motor deficits (Adamu et al., 2024).

Traditionally, research on estrogen has focused primarily on its roles in pubertal development and reproductive function. However, it is now well recognized that estrogens exert wide-ranging effects beyond reproduction. Circulating estrogens act on multiple organ systems—including the cardiovascular, immune, and central nervous systems—exerting tissue-specific biological functions. Clinical trials have shown that estrogen can alleviate brain damage caused by ischemic stroke (Zhong et al., 2023). A meta-analysis of preclinical studies suggests that estrogen helps improve morphological and cellular outcomes following neonatal hypoxia-ischemia (Durán-Carabali et al., 2023). Estrogen influences neuroinflammation not only by modulating the activation status and cytokine profiles of immune cells such as microglia, but also by affecting neuronal survival, BBB integrity, and apoptosis-related pathways (Ma et al., 2016). These multifaceted actions position estrogen as a key regulator of CNS homeostasis.

This review aims to elucidate the multi-level roles of estrogen in the regulation of neuroinflammation. We examine interconnected dimensions, beginning with neural cell responses, followed by mitochondrial regulation, DNA repair pathways and the influence of gut microbiota on neuroimmune communication. By integrating recent research findings, we seek to provide a theoretical foundation for the development of estrogen-based precision interventions.

### 1.1 Literature search strategy

We conducted a literature search in PubMed, Web of Science, and Scopus databases. The search covered articles published between January 2003and May 2025, using a combination of the following keywords: *“estrogen,” “neuroinflammation,” “estrogen receptor,” “mitochondria,” “gut-brain axis,”* and *“DNA repair.”*

*Inclusion criteria* were: (1) original research articles involving *in vitro*, *in vivo* (animal), or *clinical/observational human* studies; (2) studies specifically investigating the effects of estrogen or its receptors on neuroinflammation or related neural processes.

*Exclusion criteria* included: (1) studies unrelated to the central nervous system (CNS); (2) non-English publications; (3) studies lacking mechanistic or outcome-related data on estrogen effects.

## 2 Estrogen and estrogen receptors

Estrogen, a lipophilic steroid hormone synthesized from ovarian cholesterol, readily diffuses across membranes, including the BBB (Hao et al., 2019). Besides peripheral sources, neurons and glial cells also produce neurosteroid-derived estrogen, compensating for declining systemic levels (Saldanha, 2021). Estrogen exists as estrone, estradiol, and estriol; of these, 17β-estradiol (E2) is the most abundant, bioactive, and extensively studied in the nervous system—thus the focus of this article.

Estrogen exerts its effects through binding to estrogen receptors (ERs) via two mechanisms. The genomic pathway involves classical nuclear ERα and ERβ, which bind E2 and activate estrogen response elements on DNA to regulate target gene transcription. Different ER subtypes elicit distinct or opposing effects. Some studies suggest ERα deletion alleviates inflammation and cognitive impairment, possibly due to its promotion of NF-κB signaling. ERα may protect female rat neurons from glutamate-induced injury but shows no effect in males (Maioli et al., 2021), contributing to ongoing debate over its role. In contrast, the neuroprotective role of ERβ appears to be more clearly defined. ERβ has been found to mediate the inhibition of NF-κB-driven inflammatory pathways, oxidative stress-related factors, and the Indoleamine 2,3-dioxygenase 1-mediated tryptophan/kynurenine pathway in the hippocampus, thereby alleviating neuroinflammation. ERβ also downregulates miR-638, reducing TNF-*α*-induced pericyte migration, thus preserving BBB integrity and protecting the neurovascular unit (Kurmann et al., 2024).

With aging, the expression levels of ERα and ERβ in the brain undergo dynamic changes. In the hippocampal cornu ammonis region 1 of aged rats, both ERα and ERβ exhibit reduced synaptic expression. However, unlike ERα, ERβ can be reactivated and upregulated upon administration of E2, suggesting a selective restoration potential for ERβ (Waters et al., 2011).

The non-genomic pathway involves membrane-bound receptors rapidly activating intracellular signaling cascades. E2 can directly interact with ERα and chloride intracellular channel protein 1, enhancing the currents mediated by chloride intracellular channel protein 1 and thereby rapidly modulating the excitability of ERα-positive neurons in the brain at millisecond timescales, with broad implications for various neurophysiological processes (Yu et al., 2024). In addition to ERα and ERβ, another non-classical membrane-bound receptor, G protein–coupled estrogen receptor 1 (GPER1), also mediates estrogen signaling by activating multiple downstream pathways (PKA, ERK, PI3K), promoting the generation of intracellular cyclic adenosine monophosphate (cAMP), and regulating intracellular calcium homeostasis (Bai et al., 2020). Recent evidence further indicates that activation of GPER after global cerebral ischemia upregulates the expression of interleukin-1 receptor antagonist in the hippocampus, thereby reducing ischemia-induced cell death. By increasing interleukin-1 receptor antagonist levels in neurons, GPER enhances anti-inflammatory mechanisms and helps preserve cognitive function following global cerebral ischemia.

Estrogen receptors are widely distributed throughout the central nervous system, encompassing regions associated with higher-order brain functions such as the hypothalamus, limbic system, hippocampus, and prefrontal cortex (Fuente-Martin et al., 2013). These receptors are expressed not only in neurons but also extensively in glial cells, particularly astrocytes and oligodendrocytes. Moreover, ERs are also localized to intracellular organelles, including mitochondria, suggesting additional roles in regulating energy metabolism and apoptosis (Yang et al., 2004). The key signaling pathways, molecular targets, and functional outcomes associated with estrogen action in neural cells are detailed in Table 1.

**Table 1:** Estrogen-mediated signaling pathways and their functional outcomes in neural cells.

Target	Key signaling pathways	Major molecular targets	Functional outcomes
Astrocytes	Not specified Potential cross-talk with neurotrophins	↑ GLAST, GLT-1 ↓GFAP ↑Neurotrophic factors	Enhances glutamate uptake, reduces excitotoxicity, alleviates astrogliosis
Microglia	PI3K/Akt TLR4/NF-κB SIRT1/miR-138-5p Ferroptosis-related pathways	↑SIRT1 ↓HMGB1 ↓ATF4 ↓IL-1β	Promotes M2-like phenotype, suppresses inflammation, reduces oxidative stress
Neurons	PI3K/Akt MAPK/CREB WNT/β-catenin	↑Bcl-2, Bcl-x ↓Fas, Bax, CytC ↑ β-catenin	Inhibits apoptosis, enhances survival and plasticity, suppresses neuroinflammation
Mitochondria	PI3K/Akt AMPK/PGC-1α Nrf2/HO-1	↑COXI–III ↑Mn-SOD, GPx Stabilization of ΔΨm	Enhances OXPHOS, reduces ROS, inhibits NLRP3 inflammasome, maintains bioenergetic homeostasis
DNA Repair System	PI3K/Akt → Nrf2 BDNF signaling cascade	↑APE1 ↑Nrf2 APE1 mitochondrial/nuclear translocation	Enhances oxidative DNA repair, maintains genome integrity, protects against neurodegeneration
Gut Microbiota	Microbial metabolism of estrogen Regulation of tight junctions, mucus genes	↑Lactobacillus ↑Mucin gene expression ↑β-glucuronidase activity	Modulates estrogen bioavailability, supports gut-brain axis, reduces systemic inflammation

## 3 Estrogen and neural cells

### 3.1 Estrogen and astrocytes

Astrocytes express estrogen receptors on their surface that allow for rapid recognition and response to hormonal signaling (Rurak et al., 2021). Studies have shown that E2 can stimulate astrocytes to synthesize and release various neurotrophic factors, thereby contributing to neuroprotection (Karki et al., 2014).

In addition, E2 upregulates both mRNA and protein levels of glutamate transporters GLAST and GLT-1 in astrocytes (Pawlak et al., 2005). This enhances the capacity of astrocytes to uptake extracellular glutamate, preventing excitotoxic neuronal death caused by glutamate accumulation. In an Alzheimer’s disease model derived from induced pluripotent stem cells, studies have shown that E2 significantly alleviates the astrogliosis, which is closely related to neuroinflammation. Specifically, in a neuron-astrocyte co-culture system, E2 treatment led to a downregulation of astrocytic activation markers, such as Glial fibrillary acidic protein (GFAP), and a restoration of cell morphology to a more homeostatic state (Supakul et al., 2024). This suggests that E2 may reduce excessive astrocyte activation, thereby mitigating the inflammatory environment and helping to maintain the stability of the neuronal microenvironment.

### 3.2 Estrogen and microglia

As the principal immune cells of the central nervous system, microglia predominantly express estrogen receptors (Upadhayay et al., 2023). Under acute stress conditions such as infection or hypoxia, E2 can induce a phenotypic shift in microglia from a pro-inflammatory “M1-like” state to a reparative “M2-like” state, thereby suppressing inflammatory responses and maintaining CNS homeostasis (Thakkar et al., 2018). In chronic neuroinflammatory environments, such as those observed in neurodegenerative diseases, E2 primarily exerts neuroprotective effects by attenuating microglial neurotoxicity through ERβ and membrane-associated receptors like GPER, thus protecting neurons from sustained inflammatory damage (Loiola et al., 2019).

E2 can downregulate the expression of miR-138-5p, relieving its inhibition of the deacetylase Sirtuin 1 (SIRT1), thereby upregulating SIRT1 expression. SIRT1 further inhibits the expression of high-mobility group box 1 (HMGB1), suppressing microglial activation and the release of inflammatory factors, significantly alleviating neuroinflammation in the hippocampus (Zhang et al., 2024).

Additionally, *in vivo* and animal experiments have shown that E2 can also inhibit the ferroptosis-related factor ATF4, blocking the TLR4/NF-κB pro-inflammatory signaling pathway mediated by microglia, thereby exerting anti-inflammatory and neuroprotective effects in Parkinson’s disease models (Wang et al., 2024).

However, it is important to note that the neuroprotective effects of estrogen occur within a relatively narrow physiological concentration range. While physiological levels of E2 exert anti-inflammatory and neuroprotective functions, supraphysiological doses may exert neurotoxic effects. A recent study demonstrated this phenomenon, showing that administration of supraphysiological estradiol (sE2) at twice the physiological dose exacerbated depressive-like behaviors in ovariectomized mice. *In vitro* experiments further revealed that E2 activated the ERα/NF-κB signaling pathway in microglia, leading to a pro-inflammatory phenotype and associated neurotoxicity (Li et al., 2023). These findings suggest that the use of sE2 in estrogen replacement therapy may carry potential risks, particularly when dosing exceeds physiological levels. Therefore, rather than simply increasing E2 dosage, the development of novel compounds that specifically target estrogen receptors, particularly ERβ, may represent a more promising and safer strategy to mitigate neuroinflammation in menopausal individuals.

### 3.3 Estrogen and neurons

Estrogen exerts neuroprotective effects by modulating key signaling pathways in neurons. It activates pro-survival proteins such as PI3K, cAMP-response element binding protein (CREB), Bcl-2, Bcl-x, c-fos, and c-jun (Yune et al., 2008), while inhibiting pro-apoptotic molecules including Fas, Fas-associated protein with death domain, Bax, and the release of cytochrome C (Jia et al., 2009). Estrogen also initiates mitogen-activated protein kinase signaling, enhances CREB phosphorylation, and suppresses cell death-associated signals such as caspase-3/8 and p53, thereby promoting neuronal survival (Jover-Mengual et al., 2007).

Shakya et al. (2023) further demonstrated that E2 exerts anti-inflammatory and neuroprotective effects through activation of the canonical Wingless/Integrated (WNT) signaling pathway. This pathway involves key components such as WNT1, Frizzled receptors, Low-density lipoprotein receptor-related protein 5/6 co-receptors, and the downstream effector *β*-catenin. Chronic inflammatory stimuli are known to suppress the expression of WNT1 and *β*-catenin, leading to impaired neuronal proliferation and exacerbated cellular damage. E2 treatment reverses these alterations by upregulating WNT1 and *β*-catenin levels, thereby activating the WNT pathway, enhancing neuronal viability, and reducing inflammation-induced neurotoxicity.

## 4 Estrogen and mitochondrial function

Although the brain accounts for only about 2% of total body weight, it consumes nearly 20% of the body’s total energy, making it highly dependent on mitochondrial function (Song et al., 2024). Recent studies have demonstrated that E2 exerts neuroprotective effects in the central nervous system by enhancing mitochondrial respiration and suppressing inflammatory responses.

Upon binding to estrogen receptors, E2 further interacts with estrogen response elements located in the D-loop control region of mitochondrial DNA (mtDNA), thereby directly modulating the transcription of mitochondrial genes (Klinge, 2020). E2 has been shown to upregulate the mRNA expression of cytochrome c oxidase subunits I, II, and III (Complex IV) encoded by mtDNA (Arjmand et al., 2024; Klinge, 2008). In addition, estrogen receptor *β* (ER-*β*), present in both mitochondria and nuclei, promotes CREB phosphorylation. Phosphorylated CREB binds to the D-loop region of mtDNA, regulating the transcription of oxidative phosphorylation (OXPHOS) subunits, thus influencing the expression of mitochondrial respiratory chain proteins (Lee et al., 2008).

Under pathological conditions such as ischemia, mitochondrial reactive oxygen species (ROS) are generated, which triggers the mitochondrial translocation of the NLRP3 inflammasome and the subsequent release of mtDNA (Zhang et al., 2022). Importantly, E2 has been reported to suppress NLRP3 gene expression in the cerebral cortex under inflammatory conditions (Slowik and Beyer, 2015). Further investigations have elucidated multiple key mechanisms (Thakkar et al., 2016). Firstly, at the transcriptional level, E2 suppresses the expression of key inflammasome components, including NLRP3, ASC, caspase-1, and IL-1β, and also downregulates the expression of P2X7 and TXNIP, two well-established upstream activators of NLRP3 inflammasome activation. These findings suggest that E2 can inhibit inflammasome activation at its source by blocking the initiating signals. In support of this, recent experimental studies have demonstrated that G-1, a selective GPER1 agonist, effectively inhibits the formation of the NLRP3/caspase-1 complex and the maturation of pro-IL-1β (Bai et al., 2020). Secondly, E2 significantly impedes the assembly of the inflammasome by preventing the formation of the NLRP3–caspase-1 complex, thereby disrupting the effector phase of the inflammatory cascade. Finally, the estrogen receptor coregulator PELP1 has been shown to be essential for mediating the regulatory effects of E2 on NLRP3 inflammasome activation, highlighting the importance of ER-associated cofactors in E2-driven anti-inflammatory responses. E2 also activates protein phosphatase 2A, which inhibits NLRP3 phosphorylation and reduces downstream inflammatory signaling.

**Figure 1:**
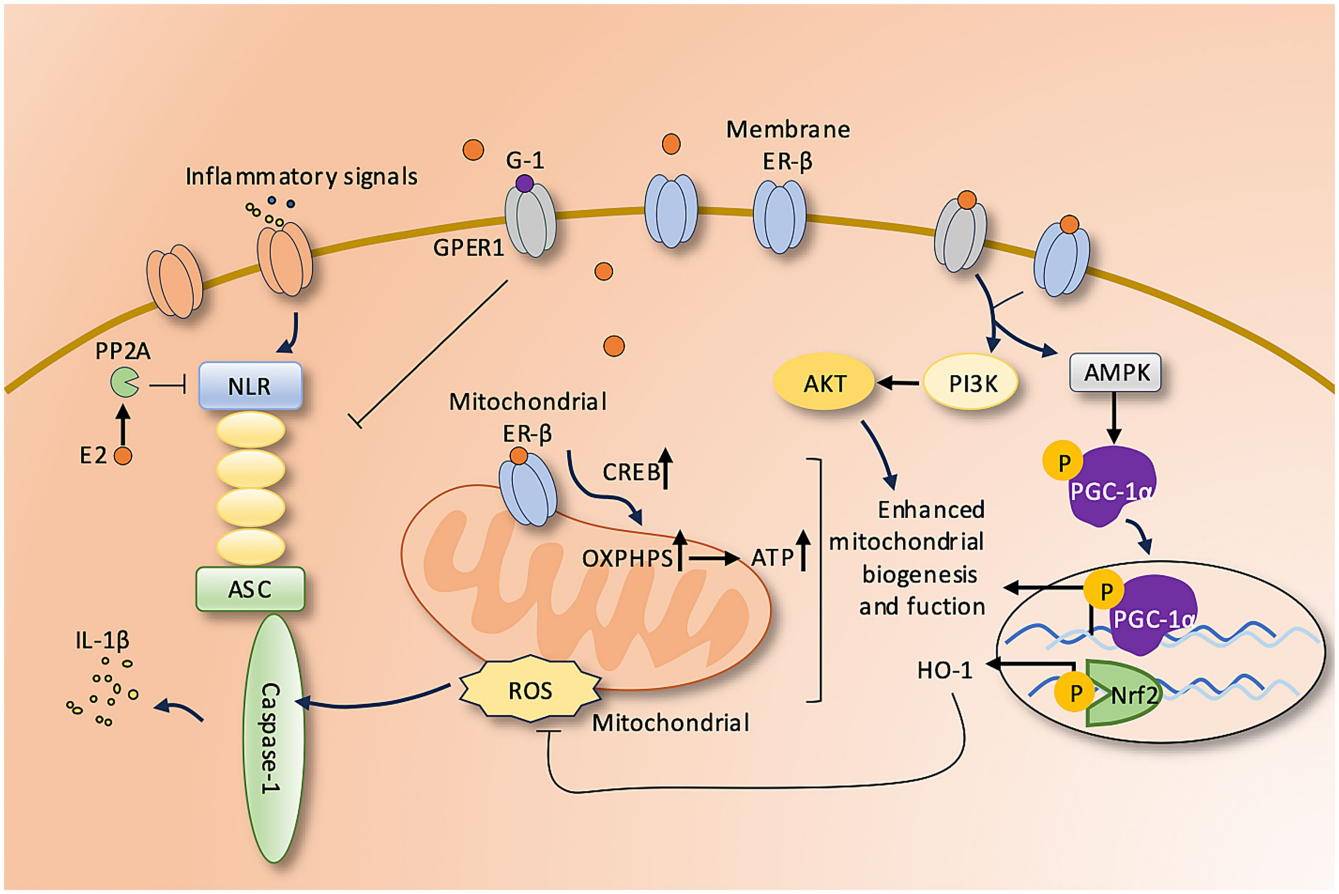
Estrogen-mediated regulation of mitochondrial function and neuroinflammation. E2 exerts neuroprotective effects through multiple pathways. Binding to membrane ER-β activates PI3K/Akt and AMPK/PGC-1α/Nrf2 signaling, enhancing mitochondrial biogenesis and antioxidant defenses. Within mitochondria, ER-β promotes CREB activation and OXPHOS, increasing ATP production and reducing ROS. E2 also inhibits NLRP3 inflammasome activation via PP2A and GPER1 signaling, thereby suppressing IL-1β release and neuroinflammation. ER-β, Estrogen receptor beta; PI3K, Phosphoinositide 3-kinase; Akt, Protein kinase B; AMPK, AMP-activated protein kinase; PGC-1α, Peroxisome proliferator-activated receptor gamma coactivator 1-alpha; Nrf2, Nuclear factor erythroid 2–related factor 2; CREB, cAMP response element-binding protein; OXPHOS, Oxidative phosphorylation; PP2A, Protein phosphatase 2A; GPER1, G protein–coupled estrogen receptor 1; NLRP3, NACHT, LRR, and PYD domains-containing protein 3; ASC, Apoptosis-associated speck-like protein containing a CARD.

Mitochondrial dysfunction—particularly characterized by ROS accumulation and the loss of mitochondrial membrane potential (ΔΨm)—is a major contributor to neuronal injury in many central nervous system disorders. E2 activates the Nrf2/HO-1 signaling pathway and upregulates key mitochondrial antioxidant enzymes, including manganese superoxide dismutase (Mn-SOD) and glutathione peroxidase (GPx), thereby enhancing mitochondrial antioxidant defenses and facilitating ROS clearance (Khan et al., 2021). In dorsal root ganglion neurons, E2 primarily activates the CaMKKβ/AMPK pathway via ERα, promoting the expression of PGC-1α and ATF3, thereby enhancing mitochondrial biogenesis and facilitating axonal regeneration (Mishra et al., 2025). In other experimental models, E2 regulates mitochondrial bioenergetics and maintains mitochondrial membrane potential (ΔΨm) through non-genomic pathways mediated by ERβ and GPER, which in turn activate downstream PI3K/Akt and AMPK/PGC-1α signaling cascades.(Guajardo-Correa et al., 2022). In traumatic brain injury models, nanomolar concentrations of E2 applied to isolated brain mitochondria significantly improved electron transport chain activity, reduced ROS production, and preserved ΔΨm in a sex-dependent manner (Kalimon et al., 2024). Figure. 1 underscores the role of E2 as a regulator of mitochondrial homeostasis.

## 5 Sex hormones and DNA repair

With aging, DNA repair capacity declines, leading to mutations in the brain (Chatterjee and Walker, 2017). While most evidence linking sex hormones to DNA repair has originated from cancer research (Zach et al., 2022), estrogens have been shown to promote DNA double-strand break repair via non-homologous end joining and improve mismatch and nucleotide excision repair (Jiménez-Salazar et al., 2021).

Though brain-focused studies are limited, estrogen protects against oxidative DNA damage by upregulating repair enzymes such as APE1 (Dietrich et al., 2013). Additionally, estrogen may indirectly enhance DNA repair capacity by inducing the expression of brain-derived neurotrophic factor (BDNF), which in turn promotes the synthesis of DNA repair enzymes such as APE1 (Scharfman and MacLusky, 2006). This establishes a neuroprotective cascade: Estrogen → BDNF → DNA repair enzymes → Enhanced DNA repair. Estrogen also activates the PI3K/Akt signaling pathway, which elevates the activity of Nrf2, a master transcription factor in the antioxidant response, further amplifying DNA repair mechanisms (Zhu et al., 2015). This pathway has been shown to confer protective effects in both brain and retinal models (Zhu et al., 2015; Ishii and Warabi, 2019).

Importantly, the influence of estrogen extends beyond gene expression to subcellular localization. For example, under estrogen stimulation, APE1 can translocate from the cytoplasm to mitochondria or specific nuclear domains, thereby enhancing regional DNA repair capacity (Rothman and Mattson, 2012). This subcellular redistribution is closely associated with oxidative stress elevation in states of estrogen deficiency, highlighting the hormone’s multifaceted role in maintaining genomic integrity and neuronal resilience.

## 6 Estrogen and the gut microbiota

Recent studies have identified a bidirectional communication system between the gut and brain—the gut–brain axis (Wang et al., 2023; Lin et al., 2024), which allows gut microbes to influence CNS function via the vagus nerve, enteric nervous system, and microbe-derived metabolites such as neurotransmitters, cytokines, and short-chain fatty acids. Estrogen, a key steroid hormone regulating neural activity, also serves as a critical mediator in this axis, modulating neuroinflammation and cognitive processes (Zim and Bommareddy, 2025).

Estrogen and the gut microbiota regulate each other reciprocally. Estrogen influences gut physiology by modulating intestinal motility, thereby altering microbial composition. Estrogen signaling enhances microbial diversity and supports the growth of beneficial bacteria like *Lactobacillus* (Zim and Bommareddy, 2025). In elderly mouse, supplementation with E2 has been found to increase the expression of mucin genes in colonic epithelial cells and improve gut barrier integrity (Song et al., 2018). Conversely, sex hormone deficiency has been shown to reduce the expression of tight junction proteins, impairing gut epithelial structure and increasing permeability. This, in turn, may facilitate the translocation of pro-inflammatory signals into systemic circulation (Song et al., 2018).

The gut microbiota contributes to systemic estrogen homeostasis. A specific subset of gut microbes, known as the “estrobolome,” is capable of metabolizing estrogens (Sui et al., 2021). Some of these bacteria produce *β*-glucuronidase, an enzyme that deconjugates bound estrogens into their active, free forms, facilitating their enterohepatic recirculation and reuse in the body (Sui et al., 2021). However, dysfunction of the estrobolome can reduce levels of bioactive estrogens, potentially contributing to metabolic disorders and neurodegenerative diseases.

This “gut–brain–estrogen axis” framework offers valuable insights into the sex-specific mechanisms underlying neurodegeneration and provides a theoretical basis for future targeted therapies.

## 7 Sex-specific effects of 17aE2 on neuroinflammation

Studies have shown that 17aE2 exerts sex-specific anti-inflammatory effects. Recent experimental evidence shows that chronic administration of 17aE2 significantly attenuates neuroinflammatory responses in male mice, characterized by reduced activation of microglia and astrocytes in both the hypothalamus and hippocampus. In contrast, this anti-inflammatory effect is not observed in female mice (Debarba et al., 2022).

Further investigation suggests that this sexual dimorphism relies on endogenous androgens like testosterone. In castrated male mice, 17aE2’s anti-inflammatory effects are significantly reduced, indicating a requirement for male sex hormones. Mechanistically, 17aE2 markedly upregulates ERα expression in the male hypothalamus, an effect absent in females (Li et al., 2023), highlighting ERα as a key mediator of its sex-specific anti-inflammatory action.

## 8 Conclusion

This review underscores estrogen’s key role in modulating neuroinflammation through multiple mechanisms. However, its diverse receptor subtype actions and sex-specific effects pose challenges, and its neuroprotection is limited to a narrow physiological range—higher doses may cause neurotoxicity, hindering clinical use.

Future research should prioritize developing ERβ-targeted selective modulators to enhance efficacy with fewer side effects. Exploring estrogen’s role in the gut–brain axis and its interaction with the microbiota also holds promise for understanding neuroinflammation and cognitive dysfunction. A deeper grasp of estrogen signaling will support more precise, personalized interventions for related diseases.

## Glossary

Akt: Protein kinase B
AMPK: AMP-activated protein kinase
ASC: Apoptosis-associated speck-like protein containing a CARD
ATF4: Activating transcription factor 4
BBB: Blood–brain barrier
BDNF: Brain-derived neurotrophic factor
CNS: Central nervous system
CREB: cAMP-response element binding protein
CytC: Cytochrome c
E2: 17β-estradiol
GFAP: Glial fibrillary acidic protein
GPER1: G protein–coupled estrogen receptor 1
GPx: Glutathione peroxidase
HMGB1: High mobility group box 1
Mn-SOD: Manganese superoxide dismutase
mtDNA: Mitochondrial DNA
NLRP3: NACHT, LRR, and PYD domains-containing protein 3
Nrf2: Nuclear factor erythroid 2–related factor 2
OXPHOS: Oxidative phosphorylation
PGC-1: Peroxisome proliferator-activated receptor gamma coactivator 1-alpha
PP2A: Protein phosphatase 2A
ROS: Reactive oxygen species
sE2: Supraphysiological estradiol
SIRT1: Sirtuin 1
WNT: Wingless/Integrated
